# Comparison of lignin derivatives as substrates for laccase-catalyzed scavenging of oxygen in coatings and films

**DOI:** 10.1186/1754-1611-8-1

**Published:** 2014-01-02

**Authors:** Kristin Johansson, Thomas Gillgren, Sandra Winestrand, Lars Järnström, Leif J Jönsson

**Affiliations:** 1 Department of Chemical Engineering, Karlstad University, SE-651 88 Karlstad, Sweden; 2 Department of Chemistry, Umeå University, SE-901 87 Umeå, Sweden

**Keywords:** Lignin derivatives, Laccase, Coating, Film, Oxygen scavenger

## Abstract

**Background:**

Lignin derivatives are phenylpropanoid biopolymers derived from pulping and biorefinery processes. The possibility to utilize lignin derivatives from different types of processes in advanced enzyme-catalyzed oxygen-scavenging systems intended for active packaging was explored. Laccase-catalyzed oxidation of alkali lignin (LA), hydrolytic lignin (LH), organosolv lignin (LO), and lignosulfonates (LS) was compared using oxygen-scavenging coatings and films in liquid and gas phase systems.

**Results:**

When coatings containing lignin derivatives and laccase were immersed in a buffered aqueous solution, the oxygen-scavenging capability increased in the order LO < LH < LA < LS. Experiments with coatings containing laccase and LO, LH or LA incubated in oxygen-containing gas in air-tight chambers and at a relative humidity (RH) of 100% showed that paperboard coated with LO and laccase reduced the oxygen content from 1.0% to 0.4% during a four-day period, which was far better than the results obtained with LA or LH. LO-containing coatings incubated at 92% RH also displayed activity, with a decrease in oxygen from 1.0% to 0.7% during a four-day period. The oxygen scavenging was not related to the content of free phenolic hydroxyl groups, which increased in the order LO < LS < LH < LA. LO and LS were selected for further studies and films containing starch, clay, glycerol, laccase and LO or LS were characterized using gel permeation chromatograpy, dynamic mechanical analysis, and wet stability.

**Conclusions:**

The investigation shows that different lignin derivatives exhibit widely different properties as a part of active coatings and films. Results indicate that LS and LO were most suitable for the application studied and differences between them were attributed to a higher degree of laccase-catalyzed cross-linking of LS than of LO. Inclusion in active-packaging systems offers a new way to utilize some types of lignin derivatives from biorefining processes.

## Background

Endeavors to decrease human impact on the climate and the environment include using resources more efficiently, changing from fossil to renewable resources, and processing resources in more efficient ways. Food production and consumption has a vast impact on climate and environment. In 2009, 230 megatons of plastics were produced worldwide, and 40% of the plastics went to packaging [1]. Since most plastics are made of petroleum, which is not a renewable resource, our massive use of plastic packages runs contrary to a sustainable development. Another environmental issue is wastage in the food-production chain. It is estimated that one third of the food produced is lost or uneaten [2]. For no good reasons, this wastage demands large amounts of electricity, fresh water, fossil fuels, and fertilizers. One possible contribution to both the package waste problem and the food wastage problem would be to use renewable and biodegradable biopolymers to produce active packages. Active packages contain active components that maintain the quality of the food that is packaged.

Lignin is a renewable biopolymer and one of the main components of wood. Although lignin is the second most abundant terrestrial biopolymer [3], it is poorly exploited and not much used in advanced material applications. Whereas the main component of processed wood, cellulose, is used for paper, paperboard, viscose and many other products, lignin often goes to energy recovery [4]. Since native lignin is recalcitrant and hard to process, it needs to be partially degraded and extracted in order to be useful. Hence, the lignin available from e.g. the pulp and paper industry is in the form of lignin derivatives. Enzymatic catalysis is an environmentally friendly way to modify the properties of chemicals and materials. Enzymes are used under mild reaction conditions and are generally highly selective catalysts. Enzymes can be utilized as catalysts of oxygen scavenging. Oxygen scavengers consume freely available oxygen in order to decrease the oxygen availability in a specified volume. In particular, oxygen scavengers can be used as an active ingredient in packaging. Food degradation processes are most often oxygen dependent, and keeping oxygen from the food is therefore a way of maintaining the quality and increasing the shelf-life of the food.

In a recent study, the enzyme laccase from the white-rot fungus *Trametes versicolor* was used to catalyze oxygen scavenging in prototype packages: coatings on cardboard and foils, and free-standing films [5]. Laccase carries out a one-electron oxidation of free phenolic hydroxyl groups present in lignin derivatives. Cross-linked products or quinones are then formed from the phenoxy radicals. In the previous investigation, lignosulfonates (LS) were used as substrate for the oxidation [5]. LS are by-products from pulp mills and wood biorefineries using sulfite-based processes. Using paperboard coatings with LS and laccase, the oxygen content in the headspace of a trial packaging was successfully reduced by up to almost 80% [5]. Furthermore, it was found that the oxidation of LS increased the storage modulus (E’) of the films, and increased their water resistance. The latter is a weak spot of biopolymer materials. The altered material properties were tentatively attributed to cross-linking, but this was not further analyzed [5].

Since interesting results were obtained with LS and laccase in coatings and films, it is of interest to compare LS with other lignin derivatives and to analyze the effects of the enzyme-catalyzed reaction on the material properties of the films. Hence, in this investigation, we also studied the oxygen-scavenging potential of alkali lignin (LA), hydrolytic lignin (LH) and organosolv lignin (LO). Alkali lignin is separated from black liquor, which is a by-product from the kraft process, by acid treatment. The kraft process is a more common industrial process than sulfite pulping, which generates LS, and the abundance of alkali lignin motivates its inclusion in the study. Hydrolytic lignin is formed by degradation of wood polysaccharides through hydrolysis, and this type of lignin may become a large co-product of lignocellulosic biorefineries [6,7]. Organosolv lignin is recovered from organosolv pulping, which is a pulping technique where organic solvents are used to extract lignin and hemicellulose. The organosolv process is studied as a pretreatment method for bioconversion of lignocellulose [8-10]. Thus, hydrolytic lignin and organosolv lignin are potential co-products of lignocellulosic biorefineries, which motivates their inclusion in the present study. Lignin co-products can be combusted for generation of energy and heat, but utilization of some part of the lignin for making high value-added products, such as active packaging, would be desirable.

The present study is focused on the properties of coated paper board, coated foil, and free-standing films containing starch and different lignin derivatives. Furthermore, by using analytical techniques such as GPC (Gel Permeation Chromatography) we investigated molecular transformations effected by laccase-catalyzed oxidation of lignin derivatives in films. Research in this area may lead to new applications for biomacromolecules such as lignin derivatives and starch, and improves the understanding of the potential of different types of lignin derivatives as well as of laccase-catalyzed reactions in solid media.

## Results and discussion

### Oxygen scavenging

The lignin derivatives LO, LA, LH and LS were evaluated as potential components of oxygen-scavenging coatings containing laccase from the white-rot fungus *Trametes versicolor* as catalyst. Initial experiments were performed to compare coatings with LO, LA and LH and to select the most promising candidate for further investigations, which also included coatings with LS.

**Figure 1:**
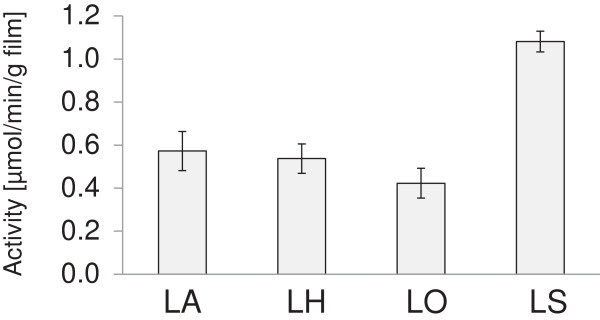
**Oxygen-consumption rate of lignin derivatives and laccase coated on aluminum foil.** Coated pieces of aluminum foil were immersed in a buffered aqueous solution (23°C and pH 6.5). The coatings contained LA, LH, LO, or LS. Error bars indicate standard deviations of three replicates.

The oxygen-consumption rate of pieces of aluminum foil coated with laccase and different lignin derivatives was determined in buffered aqueous solutions by using an oxygen electrode. Coatings with LS gave the highest value (Figure 1). Coatings with LA and LH performed similarly and reached about 50% of the oxygen-consumption rate determined for LS-containing coatings. Coatings with LO displayed slightly lower oxygen-consumption rate than the ones with LA and LH, and reached approx. 40% of that of coatings with LS. Thus, all four lignin preparations worked relatively well as substrates for laccase in coatings immersed in buffer.

**Figure 2:**
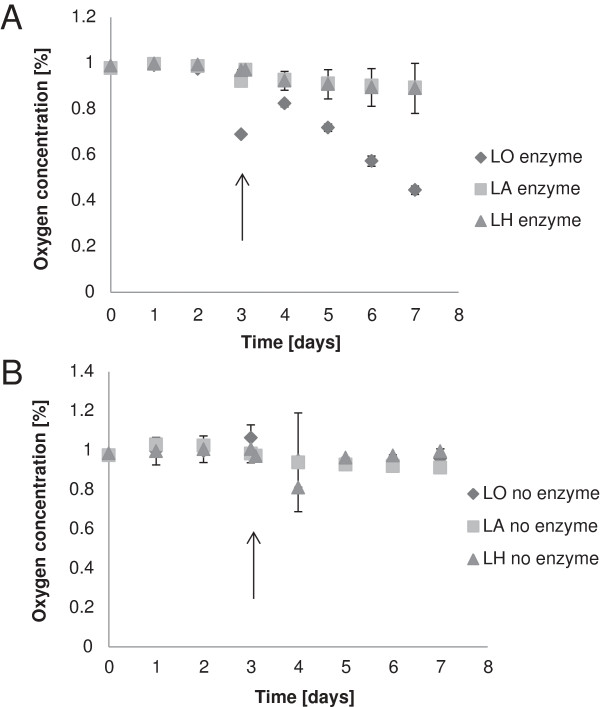
**Oxygen content in chambers with coated boards with lignin derivatives and with (A) or without (B) laccase.** The chambers were flushed with fresh gas mixture (1% oxygen and 99% nitrogen) after three days (indicated by arrows). Error bars indicate standard deviations of three replicates.

The oxygen-scavenging ability of coated boards with lignin derivatives and laccase was studied in air-tight chambers with an RH of 100% (Figure 2). As expected, coatings without enzyme did not scavenge oxygen (Figure 2B). With respect to enzyme-containing coatings, oxygen was consumed much faster by the coating containing LO than by coatings containing LA or LH. After only three days (before the re-flushing) the oxygen concentration decreased from 1% to 0.7%. This can be compared to results obtained with LS, where the oxygen concentration after three days was 0.9% [5].

**Figure 3:**
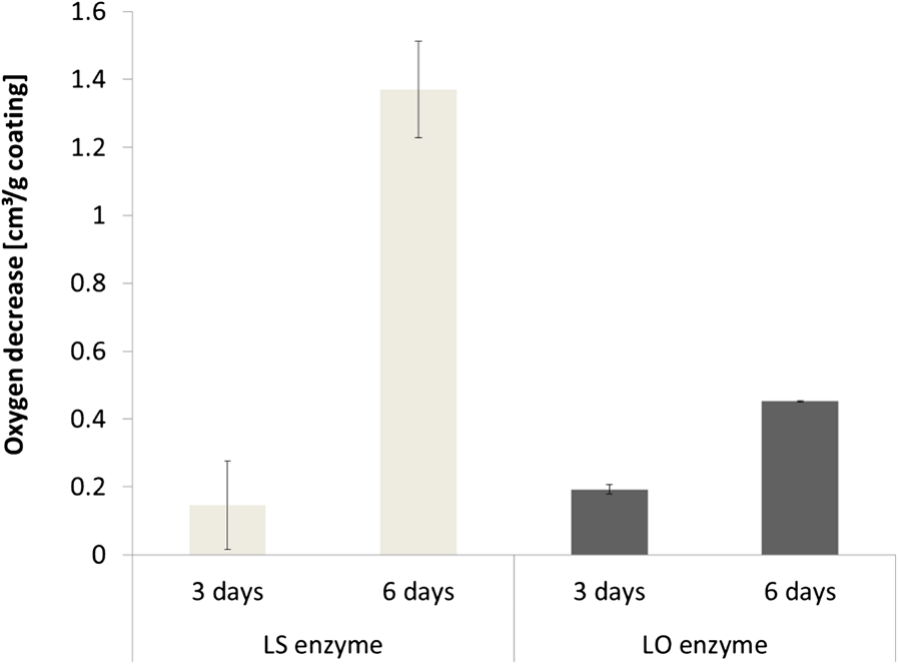
**Comparison of the amount of consumed oxygen per g of coating for LS [**[5]**] and LO (this study).** Error bars indicate standard deviations of three replicates.

After re-flushing with gas containing 1.0% oxygen and three additional days of incubation, the coating containing LS had decreased the oxygen concentration to 0.3% [5]. At day six in the experiment of the present study, three days after re-flushing with gas with 1.0% oxygen, the coating containing LO had decreased the oxygen concentration to 0.6% (Figure 2). On day seven, four days after re-flushing, the oxygen concentration was down to 0.4% (Figure 2). The coat weight of the coating containing LO was about three times as high as that of the coating containing LS, which means that for LS less substrate and enzyme was available for the reaction with oxygen. To enable a direct comparison between the LO- and LS-containing coatings, the oxygen-scavenging capacity was calculated as cm^3^ scavenged oxygen per g coating (Figure 3). The comparison indicates that after three days there was no significant difference between the LO and the LS coatings. However, after six days the coating containing LS had consumed three times more oxygen than the coating containing LO. It is likely that the plasticizing effect of water was highest for the hydrophilic LS coating, which could possibly result in a higher molecular mobility [11]. Water uptake may also explain the difference in oxygen consumption after six days between coatings with LO (Figure 2) and with LS [5]. The presence of water has been found to be of great importance for the oxygen-scavenging ability of glucose oxidase incorporated into paper coatings [12].

Since laccase oxidizes phenolic hydroxyl groups, it was of interest to see if there was a correlation between oxygen scavenging and the content of phenolic hydroxyl groups. The content of phenolic groups (given as mmol per kg of dry substance) was: LA 1390; LH 890; LS 620; LO 300. LS, which had the second lowest content of phenolic groups, showed the highest oxygen-scavenging capacity. LO, which contained the lowest content of phenolic groups, had higher oxygen-scavenging ability than both LA and LH (Figure 2). Thus, the oxygen-scavenging ability was not reflected by the content of phenolic groups in the lignin derivatives. As the coatings with LA and LH did not consume much oxygen, they were not studied further in subsequent experiments.

**Figure 4:**
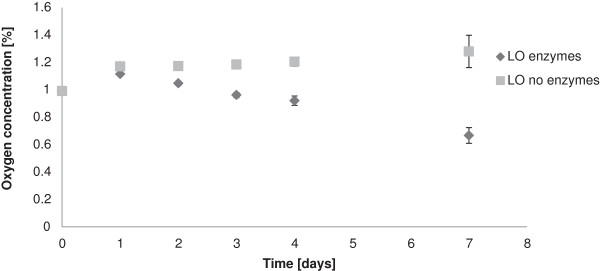
**Oxygen concentrations in air-tight chambers measured after addition of board coated with LO. The RH was 92%.** In these experiments, there was no flushing with fresh gas mixture after three days. Error bars indicate standard deviations of three replicates.

When the RH was decreased from 100% to 85%, no oxygen scavenging was detected for coatings with LS and laccase [5]. The oxygen-scavenging ability of LO-containing coatings was therefore tested using an RH in between 85% and 100%. The results are shown in Figure 4. It is clear that 92% RH was sufficient to sustain the reaction, although the rate was lower than at 100% RH. After three days at 92% RH, the oxygen concentration was still about 1.0%, and during the following four-day period it decreased to 0.7%. This result opens up for the possibility to use lignin derivatives and laccase as an oxygen-scavenging system for foodstuffs with a water activity that is higher than 0.9, which would include bread, cheese, meat, and various fruits [13].

### Laccase-catalyzed oxidation of lignin derivatives in cast starch-based films

The effects of a possible formation of a three-dimensional network upon oxidation of LO by laccase were evaluated by examining the properties of free films with respect to wet stability and mechanical properties. Films containing denatured enzyme were used as controls.

**Figure 5:**
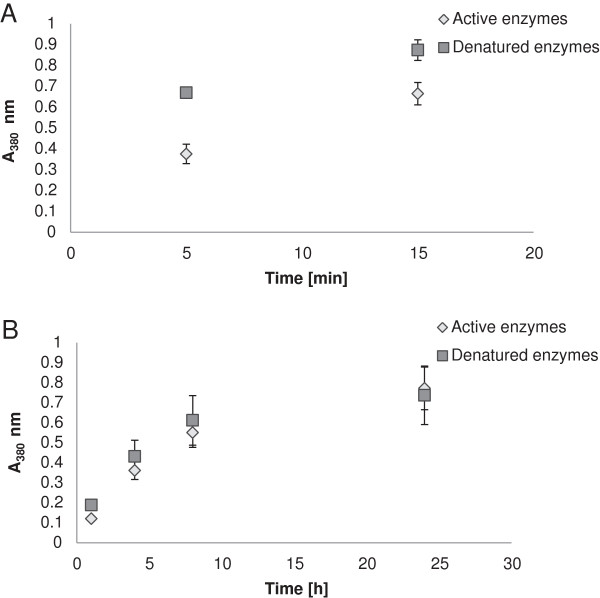
**Wet stability of free starch-based films containing LO with and without active laccase.** Samples taken early **(A)** were measured without dilution, while samples taken after a longer incubation time **(B)** were diluted 1:9. Error bars indicate standard deviations of three replicates.

Figure 5 shows the effect of active enzyme on the wet stability of free films. The absorbance at 380 nm gives a measure of the amount of LO migrating out of the film. The difference between films with active and denatured enzyme was most obvious for samples taken at an early stage (5 and 15 min) (Figure 5A). After a longer incubation, such as 24 h, there was no longer any effect of the enzymatic reaction (Figure 5B). The results indicate that there is a positive correlation between the enzymatic reaction and the wet stability of the films. The effect can be attributed to cross-linking of lignin fragments, which would lead to increased degree of polymerization and stabilization of the films. Films containing LO showed less difference between active and denatured enzyme than reported for the corresponding LS-containing films. After 24 h, the absorption was reduced by 88% in LS films containing active enzyme [5]. This observation suggests more extensive formation of three-dimensional network in the LS films than in the LO films.

### Mechanical properties

**Figure 6:**
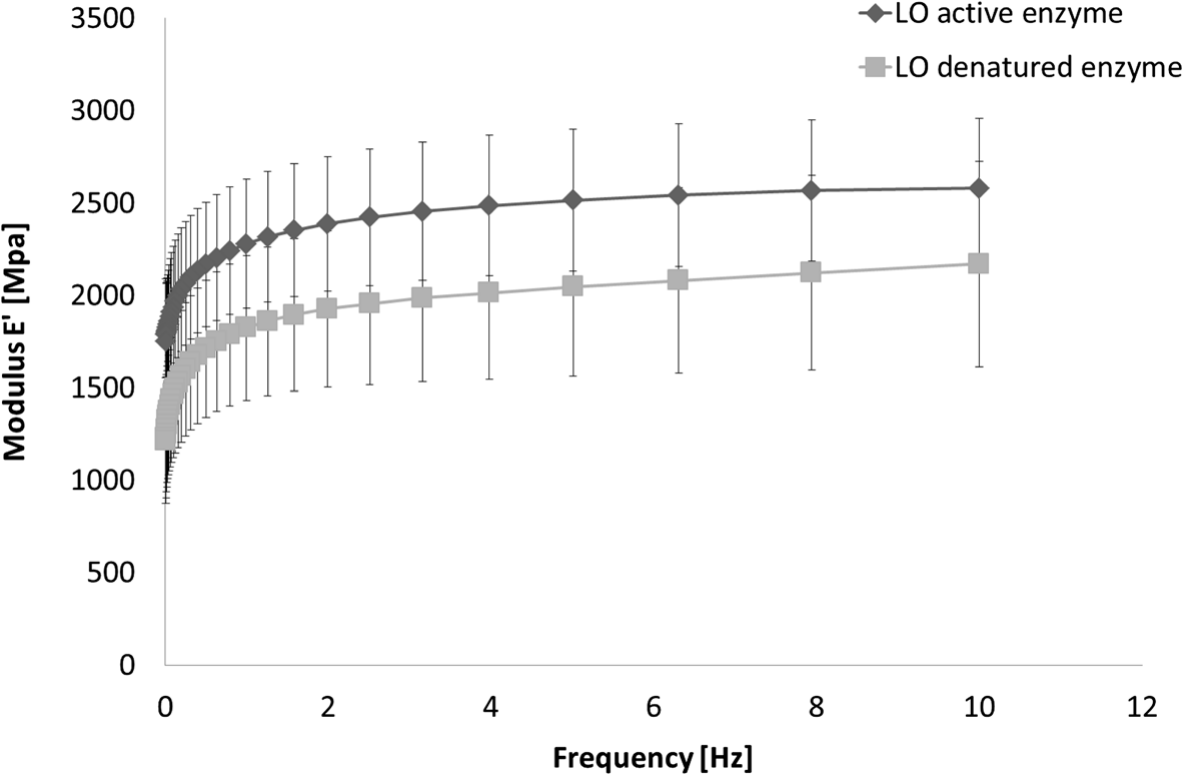
**Mechanical properties of starch-based films with LO and active or inactivated laccase.** Error bars indicate standard deviations of six replicates.

The mechanical properties of the cast starch-based films with laccase and LO were investigated by determination of the E' modulus at low frequencies. A higher E' modulus indicates that the material is stiffer with a higher cross-linking density [14]. Although the average E' modulus of the films with active laccase was higher than that of the films with inactivated enzyme, the difference was not significant (Figure 6). Similar films as these with an average thickness of 0.13 mm, with inactive enzyme but containing LS instead of LO, displayed even lower E’ modulus [5]. A plausible explanation is that LS acted as a plasticizer for the starch in the films. This explanation is supported by several investigations regarding the interaction between starch and LS [15-18]. The increase in modulus due to the active enzyme in the LS films was about 300% [5]. The results from both the mechanical testing and the wet stability testing of the starch-based films suggest that the oxidation of LO did not give rise to as extensive cross-linking as was the case for the LS-containing films. There may also be an incompatibility between the hydrophobic lignin and the hydrophilic starch. It is likely that the hydrophobic LO lignin exists as dispersed particles within the hydrophilic starch matrix [19].

Hydrophilic LS are known to be able to form complexes with starch polymers through hydrogen bonding without any oxidation reaction [20]. LS may hence facilitate the continuity of the starch network during stress, as opposed to LO which rather disrupts the starch network structure.

### Py-GC-MS analysis

The S/G (syringyl/guaiacyl) ratios of LO and LS were 2.5 and 0.012, respectively. This indicates that hardwood was used as raw material for LO, and that softwood was used for production of LS.

### GPC analysis

**Figure 7:**
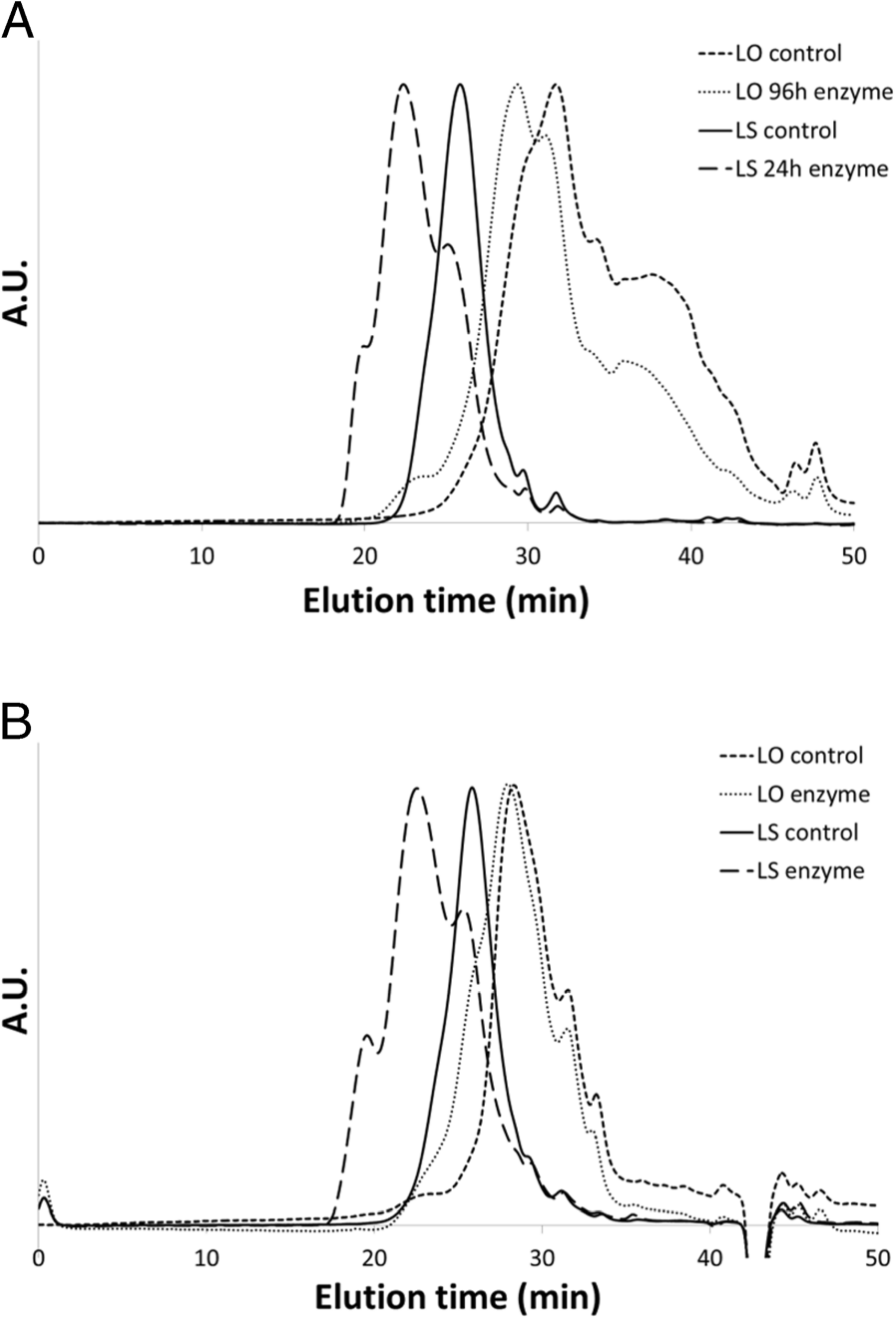
**GPC chromatogram of (A) LS and LO powder samples, and (B) LS and LO film samples.** Curves are normalized to match approximately the same maximum height.

**Figure 8:**
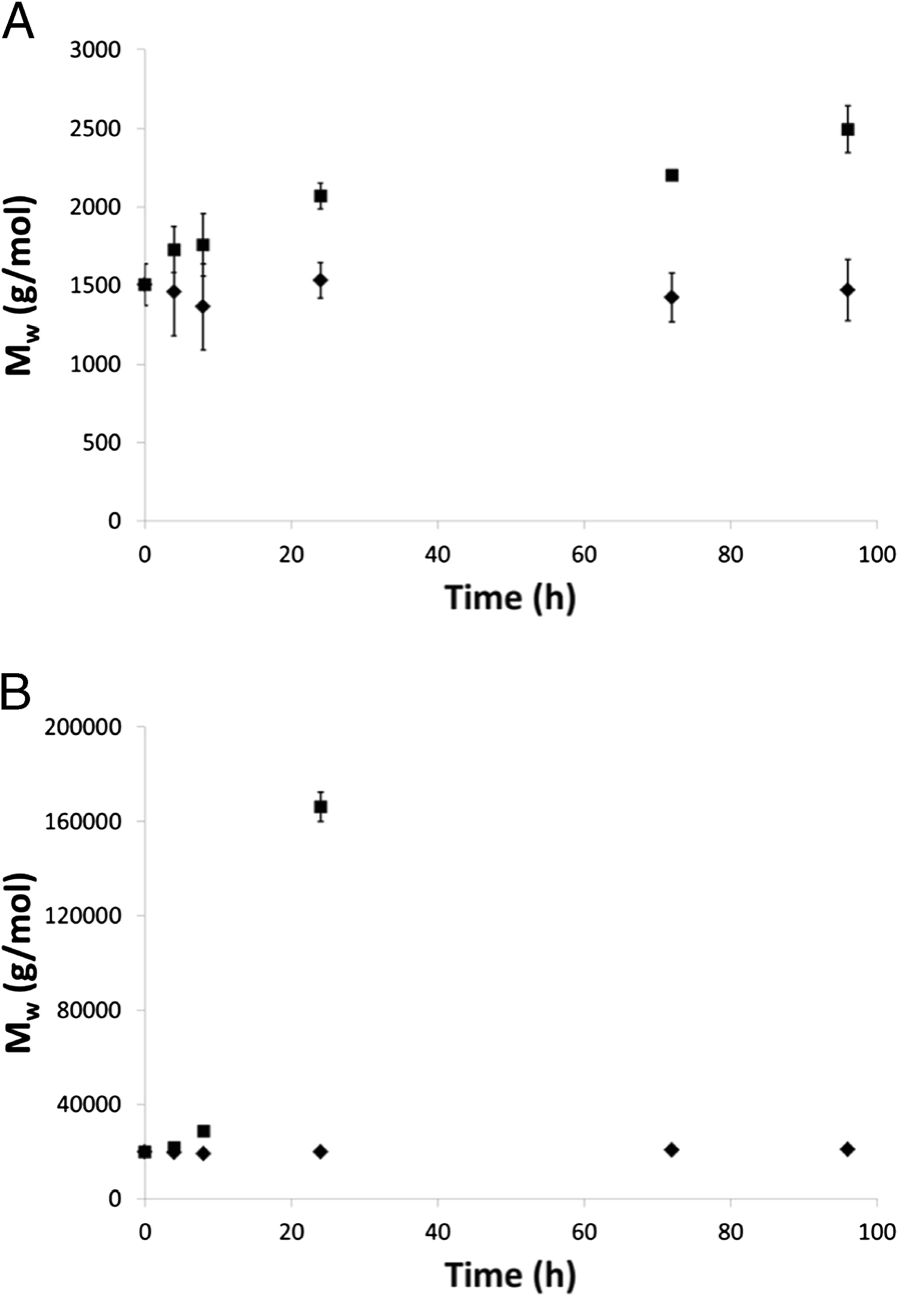
**Weight average molecular weights (M**_**w**_**) of (A) LO and (B) LS powders after incubation with active laccase (squares), and denatured laccase as controls (diamonds), at different times.** Error bars indicate standard deviations of four replicates.

Figure 7A shows the chromatograms for LO and LS freeze-dried powder samples (from reactions in solution). The main fraction of LO was divided into two peaks that were not well separated and which had very low peak molecular weights (M_p_, the most common molecular weight among the polymers in the sample) (Figure 7A). Enzymatic treatment during 96 h shifted the ratio between these peaks, as the peak with shorter elution time grew relatively the one with longer elution time. In terms of M_w_ (the weight average molecular weight) for the whole main fraction, this meant an increase from 1,500 to 2,500 as a consequence of the enzymatic activity (Figure 8). Both the enzymatically treated and the control LO powder samples showed a low-molecular-weight fraction (Figure 7A) that was absent in the films (Figure 7B). This effect was attributed to the different preparation methods used for the powder and the film samples. The initial M_p_ of the LS powder sample was 12,000, but after enzymatic treatment during 24 h it had split into three different sub-peaks with M_p_ values of 21,000, 120,000 and 620,000 (Figure 7A). The initial M_w_ was 20,000. The three sub-peaks in the analysis of the 24-h sample had M_w_ values of 13,000, 160,000, and 880,000, respectively. The reaction resulted in an eight-fold overall increase in M_w_ (Figure 8) after only 24 h. Samples with longer reaction times were not soluble and could hence not be analyzed. The lack of solubility was believed to be due to extensive cross-linking. Furthermore, according to the time courses shown in Figure 8, the molecular weight of LS increased in an exponential-like mode, which indicates that the cross-linking progressed very rapidly. In contrast, the M_w_ increase for LO was rather constant throughout the time period studied. It is worth noticing that the LS preparation possessed an initial M_w_ that was about ten-fold higher than that of LO. The lack of molecular weight increase among the control samples confirmed that it was in fact the enzyme activity that generated the molecular-weight increase of the samples incubated with active enzyme (Figure 8).

Just as for the freeze-dried LS powder samples from the oxidations reactions in water, the analysis of the LS-containing films displayed three sub-peaks with different M_p_ values (Figure 7B). The M_w_ of the sub-peaks were 7,500, 130,000, and 1,600,000, values that correspond fairly well with the M_w_ values of the LS powder samples. The M_w_ of the two sub-peaks with highest molecular weight increased roughly 9 and 110 times compared to the M_w_ of the control sample. It was most likely these high M_w_ fractions that formed the three-dimensional network responsible for the extraordinary improvements in water resistance and storage modulus of the LS-containing films.

The M_p_ of the LO film sample also increased after the enzymatic reaction (Figure 7B). The laccase-catalyzed oxidation of LO in films resulted in a five-fold increase in M_w_. Apparently, there were no high molecular weight LO fractions in the LO films. This is in agreement with the observations from the water-resistance assessment and the DMA. The more extensive cross-linking of LS (as compared to that of LO) can probably be attributed to the sulphonate groups. Since sulphonate groups make lignin more polar, they will make LS more compatible with the starch matrix. LO, on the other hand, may exist in the form of aggregates. For example, the conformation of lignin from black liquor has been found to be highly dependent on the pH in the solution, and the lignin tends to aggregate in water [21]. Compatibility with starch as a result of higher polarity of LS makes it likely that compared to LO more phenolic groups were exposed to the surroundings, and thereby they would be possible for laccase to oxidize. Furthermore, since LS is known to form complexes with starch [17], it was probably more evenly spread in the starch matrix. It has been reported that co-polymers of LS and starch can be produced through laccase-induced grafting of LS polymers onto the starch [22]. Thereby, cross-linking of LS is more likely to lead to a continuous LS network throughout the films. In contrast, LO molecules, supposedly being accumulated in aggregates, probably cross-link with other LO molecules in their vicinity. Whereas this may lead to strongly bound aggregates, it will not benefit the formation of a continuous network.

An additional contributing factor is the importance of the formation of productive couplings. Areskogh et al. [23] suggested that the presence of a sulphonate group on a lignin model compound directs the laccase-catalyzed oxidation reaction towards productive couplings, enabling further participation in cross-linking of the involved molecules. This may contribute to a more extensive cross-linking of the LS. On the other hand, oxidation of LO (which lacks sulphonate groups) may result in unproductive coupling, making further reactions more difficult. The S/G ratio may also affect the ability to form productive cross-coupling of a lignin derivative, as guaiacyl, compared to syringyl, has one additional site on the benzene moiety available for cross-linking. Further investigations are needed to unravel the roles of polarity, sulphonation and S/G content in cross-linking of lignin derivatives.

## Conclusions

Although this was not evident from experiments in buffered aqueous media, LS and LO proved to be superior to LA and LH in experiments with laccase-containing coatings incubated in oxygen-containing gas. This indicates the importance of performing this type of investigations using air-tight reaction chambers and under conditions where the humidity is well controlled. In further experiments with LS and LO, LS-containing coatings performed better with regard to water resistance and mechanical properties. GPC analysis revealed that the laccase-catalyzed reaction resulted in far more extensive cross-linking of LS as indicated by an exceptional increase in molecular weight. It seems plausible that this polymerization of LS resulted in a three-dimensional network, which was responsible for increased storage modulus and improved water resistance. The comparison suggests that sulphonation is a more important feature of lignin derivatives for making them suitable for inclusion in oxygen-scavenging coatings than high content of phenolic hydroxyl groups or low molecular weight. Another important finding is that the relative humidity does not necessarily have to be as high as 100% to obtain laccase-catalyzed oxygen scavenging with lignin derivatives, as activity was detected also at 92% RH using LO-containing coatings. This enables for use of laccase and lignin derivatives as oxygen scavengers in packages for foods such as cheese, bread, fish and fruits.

## Methods

### Materials

Laccase from the white-rot fungus *Trametes* (syn. *Coriolus*, *Polyporus*) *versicolor* was purchased from Jülich Fine Chemicals GmbH (Jülich, Germany). Technical lignin preparations [alkali lignin (LA), hydrolytic lignin (LH) and organosolv lignin (LO)] were obtained from Sigma-Aldrich (St Louis, MA, USA). The lignosulfonates (LS) were kindly provided by Domsjö Fabriker (Örnsköldsvik, Sweden). Styrene-butadiene latex (SB-latex) was supplied by Styron Europe GmbH (Horgen, Switzerland). The SB-latex had a glass transition temperature (Tg) of 6°C. The dry-solids content (SCAN-P 39:80) was 50% and the pH was 5.5. Kaolin clay (Barrisurf LX) was provided by Imerys Minerals Ltd. (Cornwall, UK). Starch (Perlcoat55, a hydroxypropylated and oxidized starch derived from potatoes) was supplied by Lyckeby Industrial AB (Kristianstad, Sweden). Glycerol (99.5%) was supplied by Karlshamns Tefac AB (Karlshamn, Sweden). Potassium nitrate and 3-(*N*-morpholino)propanesulfonic acid (MOPS) were purchased from Merck (Darmstadt, Germany) and Sigma-Aldrich, respectively. Coated boards were prepared using a three-ply packaging barrier board supplied by Stora Enso (Imatra, Finland). The board had a layer of polyethylene (PE) on the top side, and a layer of PE/ethylene vinyl alcohol (EVOH) on the reverse side. Aluminum foil (Skultuna Aluminium Folie FRYS) was obtained from Skultuna Folie AB (Skultuna, Sweden). One side of the foil is coated with polypropylene (PP). The foil was used for preparation of latex-based coatings that were used to determine the activity of immobilized enzyme. Dimethylacetamide (DMAc, HPLC grade) and lithium bromide (LiBr, ReagentPlus grade) were purchased from Sigma-Aldrich. Sodium hydroxide (NaOH) was purchased from Eka Chemicals (Bohus, Sweden). Sodium polystyrene sulfonate (PSS) standards were purchased from American Polymer Standards Corporation (Mentor, OH, USA) and Sigma-Aldrich.

### Phenolic content

The content of phenolic groups was determined by MoRe Research AB (Örnsköldsvik, Sweden) using a method based on the study by Lai et al. [24]. The relative standard error was estimated to 10%.

### Reactions with lignin derivatives in buffered aqueous solution

Reaction mixtures contained 100 mg/ml lignin preparation, 2.5 U/ml laccase, and 100 mM MOPS buffer (pH 6.5). LO was first dispersed in 100 mM alkaline MOPS buffer and subsequently adjusted to pH 6.5 using 4 M HCl. LS was added directly to the MOPS buffer (pH 6.5). One unit (U) of laccase equals the amount of enzyme required to catalyze the oxidation of 1 μmol of pyrogallol per min in a reaction performed at pH 6.5 and at a temperature of 25°C. Seven ml of the reaction mixture were transferred to five 50 ml Falcon tubes with pierced caps, and the tubes were then incubated with stirring at 23°C and 50% RH for 4, 8, 24, 72 and 96 h. The reaction mixtures were then freeze-dried (Heto Drywinner, Heto-Holten S/S, Allerød, Denmark). The 0 h sample was prepared by freeze drying seven ml of the reaction mixture immediately after enzyme addition. As controls, identical reaction mixtures as described above were prepared with the exception that the enzyme had been denatured by boiling for 12 min prior to addition. The freeze-dried samples were used for subsequent analyses using GPC (Gel Permeation Chromatography).

### Preparation of coated board and aluminum foil

A mixture of starch (20% dry-solids content) and water was prepared by vigorous stirring in a boiling water bath for 45 min. The mixture was then rapidly cooled to room temperature. The clay was dispersed in deionized water (conductivity 1 μS/cm) to a final solids content of 63%. Coating color was prepared by mixing latex, clay, freshly cooked starch, and lignin derivative in the proportions described in Table 1 (latex-based coating). The pH was adjusted to 6.5 with a 1 M solution of NaOH. The coating color was subsequently left to stir for 15 min before laccase was added (0.91 U laccase per g wet coating color). Coating color without laccase was prepared as a control. Before coating, the board was corona-treated (60 Wmin/m^2^) by using a laboratory-scale corona equipment (Corona-Plus, Vetaphone, Kolding, Denmark) to obtain sufficient adhesion between the PE-laminated board and the coating color. The treatment was performed with ceramic electrodes and an aluminum roll with a perimeter speed of 50 m/min. The corona-treated PE/EVOH-side of the board was double-coated with a bench coater (K202 Control Coater, RK Print-Coat Instruments Ltd., Royston, UK) equipped with a wire-wound bar giving a nominal wet deposit of 60 μm. The PP-side of the aluminum foil was double coated with a bar giving a nominal wet deposit of 24 μm. To ensure complete film formation, the coated board was dried at 105°C for 30 s and then at 30°C for 24 h after the first and second application of coating color.

**Table 1:** Coating-color recipes

**Component**	Latex-based coating*	Starch-based coating**
Latex	100	-
Starch	10	100
Clay	55	55
Lignin derivative	30	20
Glycerol	-	30
Enzyme preparation	6	6

*Parts of the component per hundred parts (by weight) of dry latex.

**Parts of the component per hundred parts (by weight) of dry starch.

### Casting of films

The effects of the laccase-catalyzed reaction on the water stability of the films, the mechanical properties of the films, and the size distribution of the lignin derivatives were evaluated using free films casted in Petri dishes with an inner diameter of 8.7 cm. The coating color was prepared by dispersing lignin derivatives in alkalized deionized water followed by mixing with the starch and glycerol in the proportions indicated in Table 1 (starch-based coating). Glycerol was used as a plasticizer for the starch-based films. The pH was subsequently adjusted to 6.5 using 0.5 M HCl prior to addition of the clay as it precipitates under alkaline conditions. After that, the coating color was stirred for 15 min prior to addition of laccase. A similar mixture, but containing enzyme that had been denatured through boiling for 12 min, was prepared as a control. An amount of dispersion corresponding to a total of about 1 g of dry matter was added to each Petri dish, and the films were then dried in dishes without lids for seven days at 23°C and 50% RH (relative humidity). The thickness of the films was measured prior to analysis using an STFI Thickness Tester M201 (TJT Teknik AB, Järfälla, Sweden), on average the thickness was found to be 0.12 mm.

### Oxygen scavenging in buffered aqueous solutions

The enzyme activity of coatings on aluminum foil was evaluated in a buffered solution at 25°C (20 mM MOPS, pH 6.5) using an oxygen electrode (Hansatech Ltd., King's Lynn, UK). Two cm^2^ of the coated aluminum film were weighed and added to the reaction chamber containing the pre-heated buffered solution. The decrease in oxygen concentration was monitored during 10 min and the activity was calculated as μmol oxygen/min/g film. All measurements were performed in triplicates.

### Oxygen scavenging in air-tight chambers

Two dm^2^ of board coated with latex-based coating colors with lignin derivatives were cut into strips and placed inside an air-tight chamber with a volume of 128 ml. Coated boards were analyzed in triplicates. Board coated with a coating color without enzyme was used as a control. The RH inside the chambers was varied and controlled by using a saturated salt solution of potassium nitrate giving an RH of 92% and pure water giving an RH of 100%. The RH was determined experimentally with a relative humidity tester (Testo 625, Testo GmbH & Co., Lenzkirch, Germany). The atmosphere inside the chambers was modified by flushing the chambers for 1.5 min with a gas mixture consisting of 1% oxygen and 99% nitrogen (AGA Gas AB, Enköping, Sweden). The decrease in oxygen concentration was monitored during three days before the chambers were re-flushed with 1% oxygen and the decrease was monitored for an additional period of four days. The concentration of oxygen was measured with a Checkmate II (PBI Dansensor A/S, Ringsted, Denmark) by removing an aliquot of the head-space gas for analysis using a zirconium-based sensor. The oxygen concentration was analyzed during seven days and the total decrease in oxygen concentration was calculated as cm^3^/g coating.

### Wet stability of cast starch-based films immersed in water

Cut pieces (0.6 × 1.0 cm) of films were placed in 15 ml plastic tubes containing 5 ml of the MOPS buffer. The tubes were incubated with rotation (22 rpm), and samples were taken after 5 min, 15 min, 1 h, 4 h, 8 h, and 24 h. The samples taken after 15 min were diluted 1:9 with the MOPS buffer. Following the method described by Johansson et al. [5], the migration of the lignin derivative from the film to the water phase was evaluated by measuring the increase in absorbance at 380 nm (Abs_380_) using a spectrophotometer (UV-2101PC, Shimadzu, Kyoto, Japan). Measurements were performed in triplicates.

### Mechanical properties of cast starch-based films

The films were cut into rectangular pieces (0.5 × 3.0 cm) and the thickness of each individual piece was measured using the STFI Thickness Tester. The storage modulus (E') of the films was evaluated by performing an oscillation sweep from 0.1 to 10 Hz in tension mode (DMA/SDTA861, Mettler Toledo, GmbH, Schwerzenbach, Switzerland) at constant amplitude 0.029%, 23°C, and 50% RH. An amplitude sweep was performed in order to ensure that the amplitude was within the linear region. The measurement was repeated six times for both types of samples studied (with denatured enzyme and with active enzyme).

### Gel Permeation Chromatograhy (GPC)

A 1:2 (v/v) H_2_O:DMAc mixture containing 0.1% (w/w) lithium bromide (LiBr) was used as eluent. Freeze-dried LO and LS powders prepared from samples taken after 0, 4, 8, 24, 72 and 96 h reaction were dissolved in the eluent mixture prior to analysis (1 mg sample/ml eluent). LS samples taken after 72 and 96 h were not soluble and could therefore not be analyzed. Starch-based LO and LS films were dissolved in a 10 mM solution of sodium hydroxide (20 mg film sample/ml solution) at 50°C during 7 h by shaking the samples every hour. The film samples were subsequently centrifuged during 10 min at 14,100 *g* using a MiniSpin Plus centrifuge (Eppendorf, Hamburg, Germany). Then, 75% of the liquid phase of the film samples were evacuated into a pipette and mixed with DMAc in a ratio of 3:20 (v/v). Thermogravimetric analysis (results not showed) confirmed that the solid phase of the dissolved film samples consisted of the clay incorporated in the films. The GPC system used comprised two 300 × 7.5 mm PolarGel M columns with a 50 × 7.5 mm PolarGel guard, and an ultraviolet (UV) detector (wavelength 254 nm) in a Polymer Laboratories PL-GPC 50 Plus instrument (Agilent, Santa Clara, CA, USA). The eluent flow-rate was set to 0.5 ml/min. The system was calibrated using 12 PSS standards with molecular masses ranging from 208 Da to 2,600 kDa. Two subsamples of each specimen were taken and analyzed in duplicates.

### Pyrolysis – gas chromatography – mass spectroscopy (Py-GC-MS)

The syringyl to guaiacyl (S/G) ratios of LO and LS were determined using Py-GC-MS. The analysis was performed according to a previously described method [25].

## Competing interests

The author(s) declare that they have no competing interests.

## Authors’ contribution

KJ and SW prepared coatings, films and lyophilized reaction mixtures, and carried out experiments on oxygen scavenging. KJ performed analysis of wet stability and mechanical properties. TG performed GPC analyses. LJ and LJJ supervised the work and took part in its design and coordination. LJJ conceived the study. KJ, TG and LJJ drafted the manuscript, which was read, revised and approved by all authors.

## Authors’ information

KJ is a doctoral student with focus on enzymes for applications in active packaging. TG is a postdoctoral researcher working on biopolymers from renewable resources. SW is a postdoctoral researcher working on enzyme technology in the biorefinery area. LJ is a professor with focus on paper surface treatment. LJJ is a professor with focus on biotechnology for biorefining of lignocellulosic biomass.

## References

[B1] Plastics Europe http://www.plasticseurope.org

[B2] Gustavsson J Stenberg C Sonesson U van Otterdijk R Meybeck A Global Food Losses and Food Waste 2011 Food and Agriculture Organization of the United Nations: Rome

[B3] Boerjan W Ralph J Baucher M Lignin biosynthesis Annu Rev Plant Biol 2003 8 519 46 10.1146/annurev.arplant.54.031902.134938 14503002

[B4] Dahlquist E Biomass as Energy Source: Resources, Systems and Applications 2013 Leiden: CRC Press

[B5] Johansson K Winestrand S Johansson C Järnström L Jönsson LJ Oxygen-scavenging coatings and films based on lignosulfonates and laccase J Biotechnol 2012 8 14 18 22721759 10.1016/j.jbiotec.2012.06.004

[B6] Lynd LR Laser MS Bransby D Dale BE Davison B Hamilton R Himmel M Keller M McMillan JD Sheehan J Wyman CE How biotech can transform biofuels Nat Biotechnol 2008 8 169 172 10.1038/nbt0208-169 18259168

[B7] Pu Y Kosa M Kalluri UC Tuskan GA Ragauskas AJ Challenges of the utilization of wood polymers: how can they be overcome? Appl Microbiol Biotechnol 2011 8 1525 1536 10.1007/s00253-011-3350-z 21796383

[B8] Pan X Arato C Gilkes N Gregg D Mabee W Pye K Xiao Z Zhang X Saddler J Biorefining of softwoods using ethanol organosolv pulping: preliminary evaluation of process streams for manufacture of fuel-grade ethanol and co-products Biotechnol Bioeng 2005 8 473 481 10.1002/bit.20453 15772945

[B9] Wörmeyer K Ingram T Saake B Brunner G Smirnova I Comparison of different pretreatment methods for lignocellulosic materials. Part II: Influence of pretreatment on the properties of rye straw lignin Bioresour Technol 2011 8 4157 4164 10.1016/j.biortech.2010.11.063 21208799

[B10] Panagiotopoulos IA Chandra RP Saddler JN A two-stage pretreatment approach to maximise sugar yield and enhance reactive lignin recovery from poplar wood chips Bioresour Technol 2013 8 570 577 23334012 10.1016/j.biortech.2012.12.093

[B11] Vieth WR Diffusion in and Through Polymers: Principles and Applications 1999 Münich: Hanser 73 106

[B12] Johansson K Christophliemk H Johansson C Jönsson L Järnström L The effects of coating structure and water-holding capacity on the oxygen-scavenging ability of enzymes embedded in the coating layer Tappi J 2013 8 43 52

[B13] Taoukis PS Richardson M Barbosa-Cánovas GV, Fontana AJ, Schmidt SJ, Labuza TP Water Activity in Foods: Fundamentals and Applications 2008 New Jersey: Wiley-Blackwell 273 312

[B14] Schlesing W Buhk M Osterhold M Dynamic mechanical analysis in coatings industry Prog Org Coat 2004 8 197 208 10.1016/j.porgcoat.2003.09.009

[B15] Baumberger S Lapierre C Monties B Lourdin D Colonna P Preparation and properties of thermally moulded and cast lignosulfonate-starch blends Ind Crop Prod 1997 8 253 258 10.1016/S0926-6690(97)00015-0

[B16] Lepifre S Froment M Cazaux F Houot S Lourdin D Coqueret X Lapierre C Baumberger S Lignin incorporation combined with electron beam irradiation improves the surface water resistance of starch films Biomacromolecules 2004 8 1678 1686 10.1021/bm040005e 15360275

[B17] Richardsson G Sun Y Langton M Hermansson A-M Effects of Ca- and Na-lignosulfonate on starch gelatinization and network formation Carbohyd Polym 2004 8 369 377 10.1016/j.carbpol.2004.04.023

[B18] Morais LC Curvelo AAS Zambon MD Thermoplastic starch–lignosulfonate blends. 1. Factorial planning as a tool for elucidating new data from high performance size-exclusion chromatography and mechanical tests Carbohyd Polym 2005 8 104 112 10.1016/j.carbpol.2005.03.016

[B19] Baumberger S Lapierre C Monties B Della Valle G Use of kraft lignin as filler for starch films Polym Degrad Stabil 1997 8 273 277

[B20] Baumberger S Hu TQ Starch-Lignin Films In Chemical Modification Properties and Usage of Lignin 2002 New York: Kluwer Academic/Plenum Publishers 1 20

[B21] Gidh AV Decker SR See CH Himmel ME Williford CW Characterization of lignin using multi-angle laser light scattering and atomic force microscopy Anal Chim Acta 2006 8 250 258 10.1016/j.aca.2005.09.023

[B22] Shogren RL Biswas A Preparation of starch-sodium lignosulfonate graft copolymers via laccase catalysis and characterization of antioxidant activity Carbohyd Polym 2013 8 581 585 10.1016/j.carbpol.2012.08.079 23121948

[B23] Areskogh D Nousiainen P Li J Gellerstedt G Sipilä J Henriksson G Sulfonation of phenolic end groups in lignin directs laccase-initiated reactions towards cross-linking Ind Biotechnol 2010 8 50 59 10.1089/ind.2010.6.050

[B24] Lai Y-Z Guo X-P Situ W Estimation of phenolic hydroxyl groups in wood by a periodate oxidation method J Wood Chem Technol 1990 8 365 377 10.1080/02773819008050245

[B25] Gerber L Eliasson M Trygg J Moritz T Sundberg B Multivariate curve resolution provides a high-throughput data processing pipeline for pyrolysis-gas chromatography/mass spectrometry J Anal Appl Pyrol 2012 8 95 100

